# Biological functions and clinical significance of long noncoding RNAs in bladder cancer

**DOI:** 10.1038/s41420-021-00665-z

**Published:** 2021-10-05

**Authors:** Yan Zhang, Xianwu Chen, Juntao Lin, Xiaodong Jin

**Affiliations:** Department of Urology, The First Affiliated Hospital, Zhejiang University School of Medicine, Hangzhou, Zhejiang China

**Keywords:** Bladder cancer, Tumour biomarkers

## Abstract

Bladder cancer (BCa) is one of the 10 most common cancers with high morbidity and mortality worldwide. Long noncoding RNAs (lncRNAs), a large class of noncoding RNA transcripts, consist of more than 200 nucleotides and play a significant role in the regulation of molecular interactions and cellular pathways during the occurrence and development of various cancers. In recent years, with the rapid advancement of high-throughput gene sequencing technology, several differentially expressed lncRNAs have been discovered in BCa, and their functions have been proven to have an impact on BCa development, such as cell growth and proliferation, metastasis, epithelial-mesenchymal transition (EMT), angiogenesis, and drug-resistance. Furthermore, evidence suggests that lncRNAs are significantly associated with BCa patients’ clinicopathological characteristics, especially tumor grade, TNM stage, and clinical progression stage. In addition, lncRNAs have the potential to more accurately predict BCa patient prognosis, suggesting their potential as diagnostic and prognostic biomarkers for BCa patients in the future. In this review, we briefly summarize and discuss recent research progress on BCa-associated lncRNAs, while focusing on their biological functions and mechanisms, clinical significance, and targeted therapy in BCa oncogenesis and malignant progression.

## Facts

- Bladder cancer is one of the top 10 cancers with high morbidity and mortality worldwide.
- LncRNAs are a large class of noncoding RNA transcripts longer than 200 nucleotides that play important roles in biological processes, especially in cancer progression.
- LncRNAs can regulate the progression of bladder cancer.
- LncRNAs have the potential to accurately predict BCa patient prognosis and associated with clinicopathologic characteristics.

## Open questions

- Are lncRNAs involved in the posttranscriptional regulation of bladder cancer genes?
- How can we target lncRNAs to modulate the mechanism of bladder cancer progression?
- Are more multicenter cohort studies needed to verify the clinical value of lncRNAs in bladder cancer?

## Background

As one of the most common urinary malignancies, bladder cancer (BCa) ranks within the top 10 cancers associated with high morbidity and mortality globally [1]. As a highly heterogeneous cancer, non-muscle-invasive BCa accounts for more than 75% of all BCa cases, while muscle-invasive BCa accounts for the remainders [2]. In current clinical practice, pathological biopsy with cystoscopy is considered to be the most reliable method for detecting BCa [3]. A major achievement in BCa therapies has been obtained. There is a wide range of BCa treatment plans, including surgical resection, chemotherapy, radiotherapy, and immunotherapy [4]. Despite recent progress in various cystoscopy and treatment options, the outcome of BCa patients is still not optimistic. The main reason for the low 5-year survival rate of advanced BCa patients is postoperative recurrence and uncontrollable distant metastasis [5]. Therefore, elucidating the molecular mechanisms and identifying potential therapeutic targets in BCa patients are of great significance.

The Cancer Genome Atlas (TCGA) has identified molecular aberrations at the DNA, RNA, protein, and epigenetic levels via massive numbers of human tumors analyzed. These sequencing results have confirmed that only 1–2% of human DNA is protein-coding genes, while more than 90% of the human gene (called noncoding RNAs) is transcribed to a universal team of RNA transcripts except protein-coding functions [6–8]. Long noncoding RNAs (lncRNAs), a large class of noncoding RNA transcripts, consist of more than 200 nucleotides [9]. With the rapid development of high-throughput genome sequencing technologies, lncRNAs are reported to play important roles in biological processes, especially in cancer progression, cell proliferation, differentiation, and metastasis. Several lncRNAs such as HOTAIR, PVT1, and H19, have been found to influence carcinogenesis and progression in colon cancer [9]. Recent studies have demonstrated that lncRNAs play important roles in tumor development and progression and aberrant expression of lncRNAs has been reported in BCa [10]. However, there are no studies that have systematically analyzed the role and mechanism played by lncRNAs in BCa. This review summarizes the functions and mechanisms, and clinical significance of lncRNAs in the oncogenesis and malignancy of human BCa within the last 10 years.

## Overview of lncRNAs functions in BCa

Gibb et al. suggested that the importance of lncRNAs is rising, as they play roles in the cancer paradigm demonstrating potential functions in both oncogenic and tumor-suppressive pathways [11]. The study of lncRNAs in cancer progression has gradually developed. Studies have demonstrated that the expression of lncRNAs is related to the development and progression of BCa. It has been reported that lncRNAs are engaged in the regulation of cell growth and proliferation, tumor progression, and drug chemoresistance in BCa cells (Table 1).

**Table 1:** Overview of deregulated lncRNAs in BCa.

LncRNA	Expression	Target	Functions		Ref./PMID
			Promotion	Inhibition	
AC114812.8	↑	miR-371b-5p/FUT4	Proliferation, migration, invasion, and EMT		31706102
ADAMTS9-AS2	↓			Proliferation, migration and invasion	32801743
AFAP1-AS1	↑		Proliferation and invasion		32964963
ANRIL	↑		Proliferation	Apoptosis	26449463
ARAP1-AS1	↑	miR-4735-3p/NOTCH2	Proliferation and migration	Migration	30404578
ARSR	↑	miR-129-5p/SOX4	Proliferation, migration, and invasion		31892841
ASAP1-IT1	↑		Stemness		28895409
ATB	↑	miR-126/KRAS	Proliferation, migration, and invasion		29321082
BCAR4	↑	miR-370-3p/miR-644a/TLX1	Proliferation, migration and invasion	Apoptosis	31894304 32273720
BRE-AS1	↓	STAT3	Apoptosis	Proliferation	32495865
CALML3-AS1	↑	ZBTB2/miR-4316	Proliferation, migration, and invasion		30177388
CARLo-7	↑		Proliferation, migration, invasion, and EMT		33209690
CASC11	↑	miR-150	Proliferation		30916832
CASC9	↑	miR-497-5p/FZD6 miR-758-3p/TGF-β2	Proliferation, migration, invasion, and EMT		32677984 33200222
CASC9	↑	STAT3/EZH2/PTEN	Proliferation, migration, and invasion		32982303
CCAT1	↑		Proliferation, migration and invasion		31038865
CDKN2B-AS1	↑			Gemcitabine sensitivity	29937935
CRNDE	↑		Migration and proliferation	Apoptosis	29710461
DANCR	↑	miR-149/MSI2	Proliferation, migration, and invasion		30419948
DBCCR1-003	↓	DBCCR1/DNMT1		Cell cycle, apoptosis, and DNA methylation	27777512
DDX11-AS1	↑	miR-2355-5p/LAMB3	Proliferation		32412777
DGCR5	↓	ARID1A/P21	Apoptosis	Proliferation, colony formation, cell cycle, migration, invasion, and EMT	30238982
DLEU1	↑	miR-99b/HS3ST3B1	Proliferation, invasion, and cisplatin resistance		30984249
DLX6-AS1	↑	miR-223/HSP90B1 miR-195-5p/VEGFA	Proliferation, Invasion, migration and EMT		31615303 31787849 32756011
EGFR-AS1	↑	miR-381/ROCK2	Invasion and migration		32194685
ELF3-AS1	↑	KLF8	Viability and migration		30528231
FAM83H-AS1	↑	ULK3	Proliferation, migration, invasion, EMT and angiogenesis	Apoptosis	33289601
FOXD2-AS1	↑	TRIB3/AKT/E2F1 miR-143/ABCC3	Proliferation, migration, invasion, and gemcitabine resistance		29445134 29674277
GAS5	↓	CDK6, CCL1		Proliferation and doxorubicin resistance	24069260 26548923 27878359
GAS6-AS2	↑	miR-298/CDK9	Proliferation and metastasis		30394665
GClnc1	↑	LIN28B/let-7a/MYC	Proliferation, migration, and invasion		31298933
GHET1	↑	ABCC1	Gemcitabine resistance		31115606
H19	↑	miR-29b-3p/DNMT3B EZH2/E-cad	Proliferation, invasion, migration, metastasis, and EMT		23354591 28779971
HCG18	↓	miR-34c-5p/NOTCH1	Proliferation and migration		30426533
HCG22	↓	PTBP1		Proliferation, migration, invasion and EMT	31304601
HCP5	↑	miR-29b-3p/HMGB1/TLR4	Viability, proliferation migration and invasion		33235469
HIF1A-AS2	↑	HMGA1/P53	Cisplatin resistance		30216500
HNF1A-AS1	↑		Proliferation, migration, and invasion		29541223
HOTAIR	↑	miR-205/CCNJ	Proliferation, migration and invasion	Chemosensitivity to doxorubicin and cell apoptosis	26469956 26781446
HOXA-AS2	↑	miR-125b/Smad2	Migration, invasion and stemness		30412716
HULC	↑	ZIC2	Proliferation	Apoptosis	28946549
IGFBP4-1	↑		Proliferation and cell cycle	Apoptosis	32760196
ITGB1	↑	miR-10a	Proliferation		31486485
KCNQ1OT1	↑	miR-145-5p/PCBP2 miR-218-5p/HS3ST3B1	Proliferation, migration, invasion and EMT	Apoptosis	31827399 32820233
KTN1-AS1	↑	KTN1	Proliferation, invasion, and migration		33480975
LBCS	↓	SOX2		Self-renewal and chemoresistance	30397178
LET	↓	NF90/miR-145	Gemcitabine chemoresistance and stemness		28839463
LINC00162	↑	PTTG1IP/THRAP3	Proliferation	Apoptosis and G0/G1 phase block	33344916
LINC00319	↑	miR-4492/ROMO1 miR-3127/RAP2A	Proliferation, migration and invasion		31608995 32194636
LINC00346	↑		Proliferation and migration	Cell cycle and apoptosis	28705739
LINC00460	↑		Proliferation and migration		30881506
LINC00511	↑	miR-15a-3p	Proliferation, migration, and invasion	Apoptosis	30042171
LINC00612	↑	miR-590/PHF14	Proliferation, invasion, and EMT		30940184
LINC00641	↓	miR-197-3p/KLF10/PTEN		Proliferation migration, and invasion	30060954
LINC00675	↓		Migration, invasion, and proliferation		32367602
LINC00857	↑	LMAN1	Platinum-based chemotherapy resistance		29856124
LINC01106	↑	miR-3612/ELK3 DKC1/HOXD8	Proliferation, migration, invasion, and EMT		33311496
LINC01140	↑	miR-140-5p/FGF9	Cell aggressiveness and macrophage M2 olarization		33234721
LINC01296	↑		Proliferation, cell cycle, migration, and EMT		30588032
LINC01605	↑	MMP9	Proliferation, migration, and invasion		30054424
LINC01638	↑	ROCK2	Migration and invasion		31620199
LOC572558	↓	AKT/MDM2/P53	Cell cycle arrest and apoptosis	Proliferation, migration and invasion	27130667
LSINCT5	↑	NCYM	Tumor sphere formation and EMT process		29772237
MAFG-AS1	↑	HuR/PTBP1 miR-143-3p/COX-2 miR-125b-5p/SphK1	Proliferation, migration, invasion, metastasis, and EMT		33238264 33377647 33400245
MAGI2-AS3	↓	miR-15b-5p/CCDC19 miR-31-5p/TNS1 MAGI2/PTEN		Proliferation, migration, invasion, and EMT	30442369 33104021 33231563
MALAT1	↑	miR-125b/SIRT7 miR-125b/Bcl-2/MMP-13 SUZ12 miR-124/FOXQ1 miR-101-3p/VEGF-C	Proliferation, migration, invasion, EMT, and cisplatin resistance	Apoptosis	24449823 24512850 29151968 29736319 31650173
MBNL1-AS1	↓	miR-135a-5p/PHLPP2/FOXO1 MiR-362-5p/QKI	Apoptosis	Proliferation	31769229 32194406
MEG3	↓	miR-96/TPM1 P53	Apoptosis and cisplatin chemosensitivity	Proliferation, cell cycle, migration, and invasion	23295831 29940769 30461333
MIR143HG	↓	miR-1275/AXIN2		Proliferation, cell cycle, migration, and invasion	30471109
MIR497HG	↓	E2F4		Cell growth, migration, and invasion	33363213
MIR503HG	↓		Apoptosis and cell cycle	Proliferation, cell growth, cell invasion, migration, and EMT	30672010
MNX1-AS1	↑	miR-218-5p/RAB1A	Proliferation, migration, invasion, and EMT		31843814
MORT	↓	miR-146a-5p		Migration, proliferation, and invasion	32554962
MST1P2	↑	miR-133b	Chemoresistance to DDP		32052927
MT1JP	↓	miR‐214‐3p		Proliferation, cell-cycle, and invasion	30786017
NCK1-AS1	↑	miR-143	Proliferation and stemness		32184669
NEAT1	↑	miR-410/HMGB1	Proliferation	Apoptosis	31734579
NNT-AS1	↑	miR-1301-3p/PODXL	Proliferation, migration, invasion and EMT		31782983
NRON	↑		Proliferation, migration, invasion, and EMT		32194786
OIP5-AS1	↑	OIP5	Proliferation, cell viability, and cell‐cycle	Apoptosis	30485498
OXCT1‐AS1	↑	miR-455-5p/JAK1	Proliferation and invasion		30609030
PANDAR	↑		Proliferation and migration	Apoptosis	27206339
PART1	↑		Proliferation and invasion	Apoptosis	31311442
PCAT6	↑	miR-513a-5p	Viability, migration, and invasion		33090394
PEG10	↑	miR-29b miR-134/LRP6	Proliferation, migration, and invasion	Apoptosis	30941768 30953817
PLAC2	↓	miR-663/TGF-β1	Invasion and migration		32650766
PlncRNA-1	↑	miR-136/smad3	Proliferation, migration, and invasion		33288752
PTENPL	↑	MiR-20a/PDCD4	Proliferation and migration		32271413
PVT1	↑	miR-128/VEGFC miR-194-5p/BCLAF1 miR-31/ CDK1	Proliferation, invasion and migration	Apoptosis	30076714 30317572 33188158
RMRP	↑	miR-206	Proliferation, migration, and invasion		30779067
RNF144A-AS1	↑	miR-455-5p/SOX11	Proliferation, migration, and invasion		33177836
ROR1-AS1	↑	miR-504	Proliferation and migration		31929567
RP11-79H23.3	↓	miR-107/PTEN	Apoptosis	Proliferation, migration, cell-cycle, lung metastasis, and angiogenesis	30149689
SLCO4A1-AS1	↑	miR-335-5p/OCT4	Proliferation, migration, and invasion		30863101
SNHG1	↑	miR-143-3p/EZH2	Proliferation, migration, and invasion		32885590
SNHG14	↑	miR-211-3p/ESM1	Cell cycle, colony formation, invasion, migration and proliferation	Apoptosis	33482820
SNHG16	↑	miR-98/STAT3 P21 miR-200a-3p/ZEB1/ZEB2	Proliferation, migration, invasion, and EMT	Apoptosis and cell cycle	29234154 30132983 32207096
SNHG20	↑		Proliferation, colony formation, migration and invasion	Apoptosis	30106094
SNHG3	↑	miR-515-5p/GINS2	Proliferation, migration, invasion, and EMT		32596993
SNHG5	↑	p27	Proliferation and cell cycle	Apoptosis	29434891
SNHG6	↑	miR‐125b/Snail1/2/NUAK1	Migration, invasion, and EMT		30168179
SNHG7	↑	miR-2682-5p/ELK1	Proliferation, cell viability, proliferation, cell cycle, migration, invasion, and EMT	Apoptosis	30003751 30527358 30719150 32898531
SOX2OT	↑	miR-200c/SOX2	Migration, invasion, EMT, and stemness		32019566
SPRY4-IT1	↑	miR-101-3p/EZH2	Proliferation, migration, and invasion	Apoptosis	27998761
TINCR	↑	miR-7/mTOR	Proliferation, migration, and invasion		33000269
TMPO-AS1	↑	miR-98-5p/EBF1 TMPO	Proliferation, migration and invasion	Apoptosis	32087328 32964962
TP73-AS1	↓		Apoptosis	Cell growth, cell cycle, migration, invasion, and EMT	29625110
TUC338	↑	miR-10b	Migration and invasion		31162712
TUG1	↑	miR-145/miR-142/ZEB2 miR-29c HMGB1 miR-194-5p/CCND2 Nrf2	Proliferation, migration, invasion, cisplatin resistance, radioresistance, and EMT	Apoptosis, radiosensitivity, and sensitivity of Adriamycin	26318860 28376901 28503069 29321088 30925453 31308746
UCA1	↑	miR-196a-5p/CREB C/EBPα mTOR-STAT3/miR-143 BRG1 miR-16/GLS2 miR-145/ZEB1/2/FSCN1 miR-143/HMGB1 BMP9 miR-582-5p/ATG7	Cell proliferation, migration, invasion, EMT, glycolysis, mitochondrial glutaminolysis, Cisplatin/gemcitabine resistance	Apoptosis and ROS production	22285928 24495014 24648007 24890811 24993775 26373319 26544536 27591936 28841829 29113184 29642505 30666128
UCA1a(CUDR)	↑		Proliferation, migration, and invasion	Apoptosis	22576688
XIST	↑	miR-200c miR-133a P53/TET1	Proliferation, cell clone formation, self-renewal, EMT, stemness, and migration	Apoptosis	29559853 30362292 31602223
ZEB1-AS1	↑	miR-200b/FSCN1/TGF-β1 ZEB1/AUF1	Proliferation, migration, invasion, and metastasis	Apoptosis	30823924 31115480
ZEB2-AS1	↑	miR-27b	Proliferation	Apoptosis	28992472
ZFAS1	↑	miR-329 KLF2/NKD2 ZEB1/ZEB2	Proliferation, colony formation, cell cycle, migration, and invasion	Apoptosis	29653362 29678899
ZNF503-AS1	↓	SLC8A1/GATA6	The intracellular Ca2+ concentration and cell apoptosis	Proliferation, invasion and migration	33001357
ZNFX1-AS1	↑	miR-193a-3p/SDC1	Proliferation, cell clone formation, migration, and invasion		32432735
ZNRD1-AS1	↑	miR-194/ZEB1	Proliferation, migration, invasion, and EMT		32862492

## Cell proliferation

Aberrant tumor cell proliferation can sustain active proliferative states, playing an important role in tumor growth [12]. UCA1 was the first reported oncogenic lncRNA and is overexpressed enormously in BCa and promotes BCa progression by regulating several targets and pathways [13]. First, UCA1 interferes with the chromatin redesigning activity of BRG1 and binds to the P21 promoter, thereby proliferating tumor cells [14]. The transcriptional activation of UCA1 through C/EBPα additionally contributes to elevated viability and reduced apoptosis of BCa cells [15]. Second, UCA1 regulates miR-16/GLS2 expression and suppresses ROS formation [16]. Via the mTOR/STAT3cascade and the miR143/HK2 axis, UCA1 also enhances cancer cell glucose metabolism [17]. Third, UCA1 was also reported to influence AKT expression and activity, and its alteration parallels the expression and phosphorylation of CREB to promote the proliferation and regulation of the cell cycle [18]. BMP9 upregulates AKT phosphorylation levels and increases UCA1 expression to promote the proliferation and metastasis of BCa cells [19].

The PI3K/AKT signaling pathway is the most generally activated pathway in human malignant tumors, and its activation increases the activity of nutrient transporters and metabolic enzymes to reprogram cellular metabolism inflicting tumor cell proliferation [20]. HULC promotes BCa cell proliferation via regulation of the PI3K/AKT signaling pathway and ZIC2 [21]. ATB, an oncogene, is overexpressed to promote cell proliferation and migration by regulating miR-126/KRAS via the PI3K/AKT signaling pathway [22]. FOXD2-AS1 negatively regulates the expression of TRIB3 (a negative regulator of AKT) and promotes cell proliferation, and migration [23]. However, the expression of LINC00641 is significantly decreased in BCa, and its upregulation markedly inhibits the proliferation, and metastasis of BCa cells via the PTEN/PI3K/AKT axis [24]. LOC572558 inhibits BCa cell proliferation by regulating the AKT/MDM2/P53 axis [25]. For other pathways, HCG22 negatively regulates the PTBP1-mediated Warburg effect by destabilizing human antigen R (HuR) to suppress cell proliferation, and progression [26]. IGFBP4–1 promotes cell proliferation and cell cycle progression and inhibits cell apoptosis by activating the JAK/STAT signaling pathway [27]. E2F4 is reported to be critical for MIR497HG silencing. MIR497HG suppresses cell proliferation, and metastasis by inhibiting YAP, SMAD3, BIRC5, and CCND1 expression (key genes of Hippo/Yap and TGF-b/Smad signaling) [28].

**Fig. 1:**
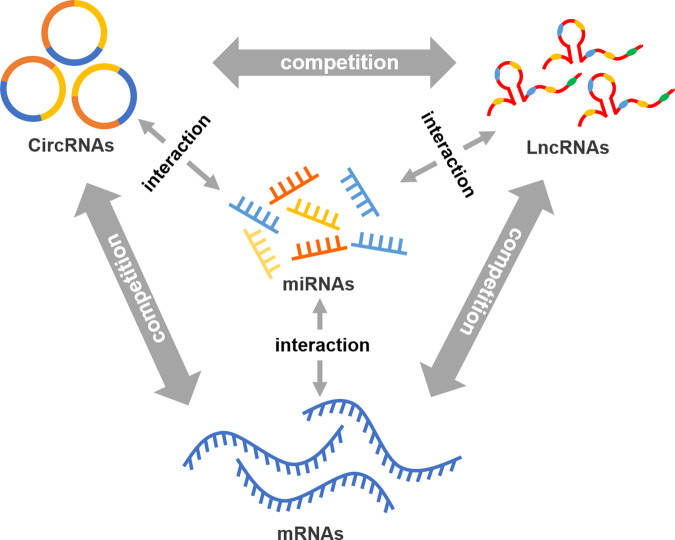
The overview of the ceRNA hypothesis.

According to various experimental studies, lncRNA functions as a competitive endogenous RNAs (ceRNAs) and competes for microRNAs (miRNAs) to regulate the expression of certain target genes (Fig. 1) [29]. The ceRNA hypothesis has become a popular method for determining the function of a large number of uncharacterized lncRNAs [30]. The ceRNA hypothesis suggests that several lncRNAs are upregulated and promote BCa progression. BCAR4 promotes the proliferation, and tumor progression of BCa cells by decreasing miR-370-3p level, and sponging miR-644a to modulate the expression of TLX1 [31, 32]. TMPO-AS1 contributes to proliferation by interacting with its sense mRNA TMPO or sponging miR-98-5p and upregulating EBF1 [33, 34]. KCNQ1OT1 has been found to regulate the miR-145-5p/PCBP2 and miR-218-5p/HS3ST3B1 axes, promote cell proliferation, and inhibit cell apoptosis [35, 36]. LINC00319 plays an oncogenic role in the regulation of proliferation and invasion by modulating the miR-3127/RAP2A and miR-4492/ROMO1 axes to regulate proliferation, migration, and invasion [37, 38]. ARAP1-AS1 promotes the proliferation and migration of BCa by regulating the miR-4735-3p/NOTCH2 axis [39]. CALML3-AS1 promotes BCa cell proliferation, and metastasis, and inhibits apoptosis by regulating the ZBTB2-mediated suppression of miR-4316 [40]. CASC11 promotes the proliferation of BCa cells by regulating miR-150 expression [41]. DANCR promotes the proliferative, migrative, and invasive ability of BCa cells by modulating the miR-149/MSI2 axis as a ceRNA [42]. GAS6-AS2 can function as a ceRNA by directly sponging miR-298 and further regulating the expression of CDK9 to promote cell proliferation and metastasis [43]. Overexpressed PVT1 downregulates miR-31 to enhance the expression of CDK1 and facilitate BCa cell proliferation, migration, and invasion [44]. SLCO4A1-AS1 promotes proliferation, migration, and invasion by sponging miR-335-5p to upregulate OCT4 expression [45]. DDX11-AS1 significantly promotes cell proliferation via the miR-2355-5p/LAMB3 axis [46]. ZNFX1-AS1 targeting miR-193a-3p/SDC1 regulates cell proliferation, migration, and invasion of BCa cells [47]. RNF144A-AS1 enhances the malignant behaviors of BCa cells via the miR-455-5p/SOX11 axis [48]. TUG1 inhibits miR-29c expression to promote cancer cell proliferation, metastasis [49]. ZFAS1 promotes cell proliferation, and metastasis by downregulating miR-329 [50]. XIST downregulates miR-133a, or P53/TET1 to promote BCa progression [51, 52]. ITGB1 promotes cell proliferation by regulating miR-10a expression [53]. ROR1-AS1 is upregulated in BCa and promotes cell growth and migration by regulating miR-504 [54]. LncRNAs can also function as inhibitors and are downregulated in BCa. MBNL1-AS1 inhibits BCa cell proliferation and enhances cell apoptosis via targeting of the miR-135a-5p/PHLPP2/FOXO1 and miR-362-5p/QKI axes [55, 56]. HCG18 suppresses cell proliferation and migration by directly sponging miR-34c-5p and regulating the expression of NOTCH1 [57]. YMT1JP suppresses cell proliferation, cell cycle progression, and invasion by regulating miR-214-3p [58].

In addition to their binding to miRNAs, some newly reported lncRNAs directly bind proteins and participate in proliferation processes. The knockdown of ZFAS1 represses BCa cell proliferation by upregulating KLF2 and NKD2 expression [59]. SNHG5 promotes BCa cell proliferation by targeting P27 [60]. GClnc1 has been shown to significantly promote cell proliferation, metastasis, and invasiveness in BCa via the LIN28B/let-7a/MYC axis [61]. Upregulation of CASC9 is induced by STAT3 to promote BCa cell proliferation, migration, and invasion by interacting with EZH2 and affecting the expression of PTEN [62]. As a tumor suppressor, GAS5 has been reported to inhibit BCa proliferation by regulating CDK6 and CCL1 expression [63, 64]. BRE-AS1 inhibits cell proliferation and accelerates cell apoptosis by mediating STAT3 expression [65]. ZNF503-AS1 can recruit transcription factor GATA6 to upregulate SLC8A1 expression, thereby increasing the intracellular Ca^2+^ concentration, repressing proliferation, and enhancing the apoptosis of BCa cells [66].

In addition, downregulation of LINC00346 inhibits BCa cell proliferation and migration, and induces cell apoptosis [67]. CRNDE strengthens cell migration and proliferation and inhibits cell apoptosis in BCa [68]. CCAT1 promotes BCa cell proliferation, migration, and invasion [69]. AFAP1-AS1 promotes the proliferation ability and invasiveness of BCa cells [70]. Overexpression of DGCR5 markedly inhibits proliferation and its ectopic expression leads to decreased BCa cell migration, invasion, and EMT, and promotes apoptosis [71].

## Cell apoptosis

Regulated cell death (RCD), also named cell suicide pathways, is of great importance in organismal development, homeostasis, and cancer pathogenesis [72]. Autophagy is an evolutionarily conserved process, in which dysfunctional cellular components are sequestered into lysosomes and degraded [73]. This process maintains cellular energy levels and promotes cellular survival. LncRNAs are reported to modulate autophagy [74]. ADAMTS9-AS2 inhibits BCa progression by affecting several key autophagy and apoptotic proteins [75]. Similarly, a study by Ying et al. demonstrated that insufficient expression of MEG3 could activate autophagy and promote cell proliferation [76]. Another study by Liu et al. showed that low-expression of MEG3 inhibits apoptosis of BCa by regulating miR-96 along with TPM1 [77]. In contrast, UCA1 targets miR-582-5p and promotes BCa invasion, migration, growth, and drug resistance through ATG7-mediated autophagy inhibition [78].

Numerous studies indicate that the activity of Wnt/β-catenin signaling can either foster or restrain the processes of apoptosis based on specific cellular environmental stimuli [79, 80]. Low TUG1 expression inhibits BCa cell proliferation and induces apoptosis by promoting ZEB2 mediated miR-142 suppression via inactivation of the Wnt/β-catenin pathway [81]. LINC00511 knockdown suppresses the proliferation and promotes apoptosis of BCa cells by suppressing the activity of the Wnt/β-catenin signaling pathway [82]. Cao et al. showed that SNHG16 is overexpressed in BCa tissues and cell lines and can notably promote proliferation by suppressing apoptosis of BCa cells by targeting P21 expression and regulating the miR-98/STAT3/Wnt/β-catenin axis [83, 84].

Increasing evidence suggests that lncRNAs can affect cell apoptosis by regulating the miRNA-mRNA axis or directly targeting gene expression. As a target of miR-125b, MALAT1 is upregulated in BCa and inhibits BCa cell apoptosis by regulating Bcl-2/MMP-13 and SIRT7 [85, 86]. Another study by Shan et al. showed that NEAT1 inhibits cell apoptosis by regulating miR-410 mediated HMGB1 expression [87]. SNHG14 increases the growth and metastasis of BCa and inhibits apoptosis by regulating the miR-211-3p/ESM1 axis [88]. LINC00162 can regulate PTTG1IP expression by binding THRAP3 to promote cell proliferation and inhibit apoptosis [89]. Other lncRNAs, such as SNHG7 [90, 91], ANRIL [92], ZEB2-AS1 [93], OIP5-AS1 [94], and PART1 [95], also have the same effects.

## Invasion, migration, and metastasis

Tumor cells can invade peripheral tissues and spread to the circulatory system or lymphatic system through invasion, migration, and metastasis, leading to the colonization of distant organs [96]. LncRNAs have been reported to play critical regulatory roles in tumor progression. The Wnt/β-catenin signaling pathway also plays a crucial role in invasion, migration, and metastasis [79]. LncRNAs promote tumor progression via the Wnt/β-catenin signaling pathway. Overexpression of H19 increases BCa migration and metastasis by interacting with EZH2 and downregulating E-cadherin expression through Wnt/β-catenin pathway activation [97]. Numerous studies have reported that H19 functions as a ceRNA that leads to EMT and metastasis of BCa via the miR-29b-3p/DNMT3B axis [98]. DLX6‑AS1 promotes cell proliferation, invasion, and migration in BCa by modulating the miR-223/HSP90B1 and miR-195-5p/VEGFA axes, and the Wnt/β‑catenin signaling pathway [99–101]. CASC9 positively regulates FZD6 expression by sponging miR-497-5p and subsequently activates the Wnt/β-catenin signaling pathway to promote cell metastasis [102]. Downregulated SNHG7 inhibits cell proliferation and migration in BCa by regulating the miR-2682-5p/ELK1/Src/FAK axis and activating the Wnt/β-catenin pathway [103, 104]. PEG10 as an oncogene in BCa facilitates cell growth, migration, and invasion by mediating the miR-29b and miR-134/LRP6 axis to activate the Wnt/β-catenin and JAK/STAT or JNK signaling pathways [105, 106]. PVT1 can regulate the miR-128/VEGFC and miR-194-5p/BCLAF1 axes to promote metastasis by activating the Wnt/β-catenin pathway [107, 108]. NNT-AS1 enhances cell proliferation, migration, and invasion by regulating the miR-1301-3p/PODXL axis and activating the Wnt pathway [109]. SNHG20 promotes cell proliferation, and metastasis by activating the Wnt/β-catenin signaling pathway [110]. Some tumor suppressor lncRNAs can inhibit BCa development by the Wnt pathway, such as MIR143HG, which can modulate the miR-1275/AXIN2 axis [111]. LINC00675 regulatesβ-catenin expression and is associated with BCa cell migration, invasion, and proliferation [112].

Increasing evidence suggests that ceRNAs play an important role in BCa metastasis mechanisms. ZEB1-AS1 regulates the miR-200b/FSCN1 axis and enhances migration and invasion induced by TGF-β1 in BCa cells [113]. Zhao et al. demonstrated that ZEB1‑AS1 also induces migration and metastasis via AUF1-mediated translation activation of the ZEB1 mRNA mechanism [114]. Silencing of TINCR expression significantly reduces BCa cell proliferation, migration, and invasion by regulating miR-7 and mTOR expression [115]. HOTAIR promotes the proliferation, migration, and invasion of BCa cells by regulating CCNJ and inhibiting miRNA-205 [116]. MAFG-AS1 regulates the miR-125b-5p/SphK1 and the miR-143-3p/COX-2 axes to promote the proliferation, migration, and invasion of BCa cells [117, 118]. SPRY4-IT1 sponges miR-101-3p to promote the proliferation, migration, and invasion of BCa cells by upregulating EZH2 [119]. OXCT1-AS1 promotes cell invasion via the miR-455-5p/JAK1 axis [120]. EGFR‑AS1 may promote cell invasion and migration by regulating the miR-381/ ROCK2 axis in BCa [121]. HCP5 promotes cell invasion and migration by sponging miR-29b-3p and regulating HMGB1 and TLR4 expression [122]. LINC01140 can regulate miR-140-5p/FGF9 axis as ceRNA to modulate the BCa phenotype, affect macrophage M2 polarization through the tumor microenvironment, and affect BCa cell aggressiveness [123]. In contrast, MAGI2-AS3 and PLAC2 are downregulated in BCa. MAGI2-AS3 can regulate miR-15b-5p/CCDC19 and miR-31-5p/TNS1 to inhibit proliferation, migration and invasion [124, 125]. PLAC2 suppresses BCa cell metastasis by targeting the miR-663/TGF-β1 axis [126].

ZFAS1 knockdown inhibits cell migration and invasion by downregulating ZEB1/ZEB2 expression [59]. TUC338 promotes metastasis but not the proliferation of BCa and positive expression of miR-10b [127]. ELF3-AS1 increases the viability and migration of BCa cells by interacting with KLF8 and increasing MMP9 expression [128]. A higher level of LINC01638 expression promotes the migration and invasion of BCa cells and increases ROCK2 expression [129]. PCAT6 promotes the viability, migration, and invasion of BCa cells by targeting miR-513a-5p [130]. Low expression of MORT induces cell invasion, migration, and proliferation by upregulating miR-146a-5p [131]. RMRP promotes the proliferation, migration, and invasion of BCa via miR-206 [132]. Other overexpressed lncRNAs, including HNF1A‑AS1 [133], PANDAR [134], and LINC00460 [135], can promote the migration and/or invasion of BCa.

## EMT process

The EMT process is defined as the transformation process of epithelial cells to mesenchymal cells, providing cells with the ability to metastasize and invade. UCA1 regulates the miR-143/HMGB1 axis, and promotes the invasion and EMT of BCa cells [136]. Similarly, SNHG3 promotes the EMT process through the miR-515-5p/GINS2 axis [137]. ZNRD1-AS1 knockdown inhibits cell metastasis, and EMT of BCa by regulating miR-194/ZEB1 [138]. The EMT process of BCa cells partly relies on SNHG16 via the miR-200a-3p/ZEB1/ZEB2 axis [139]. SNHG6 promotes cell metastasis and EMT partly by targeting the miR-125b/Snail1/2/NUAK1 axis [140]. MALAT1 knockdown inhibits TGF-b–induced EMT and is associated with SUZ12 [141]. It also assists tumor growth and metastasis by targeting the miR-124/FOXQ1 axis [142]. MNX1-AS1 promotes the proliferation, metastasis, and EMT process of BCa by targeting miR-218-5p/RAB1A expression [143]. LINC00612 enhances BCa cell invasion and EMT by sponging miR-590/PHF14 expression [144]. AC114812.8 promotes cell proliferation, migration, invasion, and EMT through the miR-371b-5p/FUT4 axis [145]. ARSR sponges miR-129-5p to promote proliferation, migration, invasion, and EMT processes by increasing SOX4 expression [146]. LINC01116 increases the expression of ELK3 by adsorbing miR-3612 and stabilizes HOXD8 mRNA by binding with DKC1. With the combination of ELK3 and HOXD8, LINC01116 promotes cell proliferation, metastasis, and the EMT process [147].

Furthermore, lncRNAs can also regulate the EMT process via some signaling pathways. CASC9 sponges miR‑758‑3p/TGF-β2 (a key gene of the TGF-β signaling pathway) expression to promote proliferation and EMT [148]. LSINCT5 activates Wnt/β-catenin signaling by interacting with NCYM to promote the EMT process [149]. CARLo-7 enables the proliferation, metastasis, and EMT of BCa cells by regulating the Wnt/β-catenin and JAK2/STAT3 signaling pathways [75].

LncRNAs can directly regulate target gene expression and affect the EMT process. Overexpression of MAGI2-AS3 inhibits EMT by regulating the MAGI2/PTEN axis [150]. MIR503HG inhibits cell growth, metastasis, and EMT in BCa [151]. P73-AS1 inhibits cell growth, and cell metastasis, and promotes cell apoptosis. In addition, P73-AS1 blocks the EMT process by inhibiting VIMENTIN, Snail, MMP2, and MMP9 expression and upregulating the expression of E-cadherin [152]. In contrast, MAFG-AS1 promotes proliferation, invasion, metastasis, and EMT via regulation of the HUR/PTBP1 axis [153]. LINC01605 upregulates the expression of matrix MMP9 to promote cell proliferation, migration, and invasion by activating the EMT pathway [154]. LINC01296 [155] and NRON [156] also promote the EMT process in BCa.

## Angiogenesis

Angiogenesis plays a critical role in tumorigenesis and the diffusion of malignant lesions by enhancing nutrient and oxygen supplies as well as providing a conduit for distant metastasis [157]. FAM83H-AS1 binds to c-Myc-mediated ULK3 to activate the Hedgehog signaling pathway, and FAM83H-AS1 knockdown inhibits the expression of CD31 and VEGFA (indicators of angiogenesis), suggesting that FAM83HAS1 promotes growth, metastasis, and angiogenesis of BCa cells through ULK3 upregulation and hedgehog activation [158]. In contrast, downregulation of RP11-79H23.3 led to higher CD31 and S100A4 expression and more microvessels. Moreover, RP11-79H23.3 can regulate the expression of the miR-107/PTEN axis and activate the PI3K/AKT signaling pathway to contribute to the proliferation, migration, apoptosis, and angiogenesis of BCa cells [159].

## Chemoresistance and radio-resistance

As a first-line treatment for BCa in clinical practice, chemotherapy reduces tumor masses in most patients. However, most patients gradually become unresponsive after multiple treatment cycles and eventually suffer tumor recurrence [160]. Several lncRNAs have been shown to modify the chemotherapy response in BCa. Cisplatin, a basic drug of first-line treatment for chemotherapy, is shown to significantly improve the prognosis in sensitive patients [161]. As an oncogene, TUG1 induces the expression of EZH2 and directly sponges miR-194-5p. Low levels of miR-194-5p result in increased expression of CCND2, which promotes the chemoresistance of BCa cells to cisplatin [162]. Moreover, TUG1 knockdown enhances the sensitivity of BCa cells to adriamycin [163]. LINC00857 knockdown sensitizes BCa cells to cisplatin, by negatively regulating the target gene LMAN1, indicating that LINC00857 can regulate sensitive patient responses to platinum-based chemotherapy [164]. In cisplatin-resistant BCa cells, a high level of HIF1A-AS2 enhances the expression of HMGA1 to constrain the transcriptional activity of p53 family proteins, which affects cisplatin-induced apoptosis [165]. A previous study reported that DLEU1 enhances cisplatin resistance by competitively regulating miR-99b and restoring the expression of the target gene HS3ST3B1 [166]. Downregulated MALAT1 enhances the cisplatin sensitivity of BCa cells via the miR-101-3p/VEGFC axis [167]. MST1P2 has been found to regulate the miR-133b/SIRT1 axis and suppress the sensitivity of BCa cells to cisplatin [168]. UCA1 decreases the cisplatin sensitivity of BCa cells by enhancing the expression of Wnt6 [169]. lncRNAs can also inhibit drug resistance and promote the chemosensitivity of BCa cells to cisplatin. For example, overexpression of MEG3 sensitizes BCa cells to the chemotherapy drug cisplatin [170].

Gemcitabine is another cytotoxic chemotherapeutic agent of BCa cells, but the majority of patients, similar to those treated with cisplatin, ultimately experience tumor recurrence [171]. The upregulation of LET hinders BCa recurrence when treating with gemcitabine. However, the proinflammatory cytokine TGFβ1 can directly decrease LET expression levels in gemcitabine-resistant patients [172]. However, FOXD2-AS1 positively regulates ABCC3 protein via miR-143 targeting, and its knockdown suppresses the 50% inhibitory concentration of gemcitabine, the expression of drug resistance-related genes (MDR1, MRP2, LRP1), invasion, and ABCC3 protein expression in gemcitabine-resistant BCa cells [173]. High-expression levels of CDKN2B-AS are related to low gemcitabine sensitivity, and downregulated CDKN2B-AS gene levels inactivate the Wnt signaling pathway and ultimately affect the sensitivity of BCa cells to gemcitabine [174]. Similarly, the high expression of GHET1 is associated with low gemcitabine sensitivity in BCa patients, and knockdown of GHET1 advances gemcitabine-induced cytotoxicity [175]. In addition, UCA1 activates the transcription factor CREB, by binding with its promoter and leading to miR-196a-5p expression, while knockdown of UCA1 decreases chemosensitivity to cisplatin/gemcitabine by inhibiting BCa cell growth [176].

More investigations have revealed that lncRNAs also play an important role in chemosensitivity to doxorubicin in BCa. HOTAIR overexpression promotes cell proliferation and inhibits chemosensitivity to doxorubicin, while cell apoptosis is induced by doxorubicin, and GAS5 enhancement reduces chemotherapy resistance to doxorubicin [177, 178].

For the radioresistance of BCa, the miR-145/ZEB2 axis mediates TUG1 function in EMT and radioresistance, and TUG1 downregulating increases radiosensitivity in BCa by inhibiting the targeting gene HMGB1 [179, 180].

## BCa stem cells

Although both cancer stem cells (CSCs) and normal tissue stem cells possess the abilities to undergo self-renewal and differentiation, self-renewal is typically deregulated in CSCs [181]. LncRNAs have been reported to regulate cellular identity and differentiation in cancer. Depletion of ASAP1-IT1 in T24 cells reduces the CD44 population, whereas forced overexpression of ASAP1-IT1 in J82 cells enhances cancer cell stemness, suggesting that ASAP1-IT1 is sufficient and necessary for the maintenance of stemness [182]. Overexpression of NCK1-AS1 reduces miR-143 expression and promotes proliferation and increases CD133 expression [183]. HOXA-AS2 is upregulated in BCa cells and Wang et al. reported that it is positively correlated with the expression of OCT4. In addition, HOXA-AS2 promotes the migration, invasion, and stemness of BCa cells [184]. SOX2OT is highly expressed in BCa, upregulates SOX2 expression by sponging miR-200c, and downregulates SOX2OT to inhibit BCSC self-renewal, cell migration, invasion, and EMT [185]. LBCS can inhibit BCSC self-renewal and chemoresistance by suppressing SOX2 expression [186].

## LncRNAs are associated with clinicopathological characteristics

Numerous reports show that lncRNAs have two main functions in promoting or inhibiting tumor development. Further analysis has shown that lncRNAs are closely related to many clinicopathological characteristics, such as stage, tumor size, and grade (Table 2).

**Table 2:** Relationship between LncRNAs level and clinicopathologic characteristics in BCa.

Year	Author	LncRNA	Expression	Sample	Age	Tumor size	Grade	TMN	Stage	Ref./PMID
2013	Han et al.	MALAT1	↑	27			√		√	24512851
2015	Tan et al.	TUG1	↑	54				√		26318860
2015	Chen et al.	n336928	↑	95			√		√	26551459
2016	Shang et al.	HOTAIR	↑	35			√			26781446
2016	Zhan et al.	PANDAR	↑	55			√	√		27206339
2016	Qi et al.	DBCCR1-003	↓	24			√			27777512
2017	Zhang et al.	GAS5	↓	82			√			27878359
2017	Liu et al.	SPRY4-IT1	↑	60			√	√		27998761
2017	Lv et al.	H19	↑	35				√		28779971
2017	Yang et al.	ASAP1-IT1	↑	58				√		28895409
2017	Wang et al.	HULC	↑	276					√	28946549
2017	Wu et al.	ZEB2-AS1	↑	52		√		√	√	28992472
2017	Cao et al.	SNHG16	↑	46				√	√	29234154
2018	Li et al.	CASC2a	↓	112		√		√		29358570
2018	Ma et al.	SNHG5	↑	67		√		√	√	29434891
2018	Su et al.	FOXD2-AS1	↑	100					√	29445134
2018	Tuo et al.	TP73-AS1	↓	128				√	√	29625110
2018	Yang et al.	ZFAS1	↑	102				√	√	29678899
2018	Cheng et al.	CRNDE	↑	54				√		29710461
2018	Jiao et al.	MALAT1	↑	56					√	29736319
2018	Wang et al.	HNF1A-AS1	↑	191			√	√		29762827
2018	Zhu et al.	LSINCT5	↑	108		√		√		29772237
2018	Xie et al.	CDKN2B-AS1	↑	81			√			29937935
2018	Zhong et al.	SNHG7	↑	134		√		√	√	30003751
2018	Qin et al.	LINC01605	↑	92			√		√	30054424
2018	Zhao et al.	SNHG20	↑	54				√		30106094
2018	Liu et al.	n346372	↑	60			√		√	30365104
2018	Avgeris et al.	GAS5	↓	363			√			30374124
2018	Chen et al.	LBCS	↓	120			√		√	30397178
2018	Wang et al.	HOXA-AS2	↑	80				√	√	30412716
2018	Zhan et al.	DANCR	↑	106			√	√		30419948
2018	Liu et al.	MEG3	↓	45			√	√		30461333
2018	Xie et al.	MIR143HG	↓	42			√	√	√	30471109
2018	Wang et al.	OIP5-AS1	↑	112		√		√	√	30485498
2018	Shan et al.	FAM83H-AS1	↑	96				√	√	30537032
2018	Qiu et al.	MIR503HG	↓	70			√	√	√	30672010
2019	Chen et al.	SNHG7	↑	92		√		√	√	30527358
2019	Guo et al.	ELF3-AS1	↑	102				√		30528231
2019	Wang et al.	LINC01296	↑	78			√	√	√	30588032
2019	Xu et al.	SNHG7	↑	72			√	√		30719150
2019	Cao et al.	RMRP	↑	91		√		√		30779067
2019	Yang et al.	SLCO4A1-AS1	↑	58				√	√	30863101
2019	Yu et al.	TUG1	↑	87				√		30925453
2019	Zhang et al.	CCAT1	↑	34		√	√		√	31038865
2019	Li et al.	GHET1	↑	74			√	√		31115606
2019	Zhuang et al.	GClnc1	↑	60				√		31298933
2019	Jiang et al.	HCG22	↓	78		√		√	√	31304601
2019	Dai et al.	ITGB1	↑	36					√	31486485
2019	Zhou et al.	XIST	↑	52				√		31602223
2019	Yang et al.	LINC00319	↑	47					√	31608995
2019	Wei et al.	MBNL1-AS1	↓	21		√			√	31769229
2020	Liao et al.	ARSR	↑	62		√	√			31892841
2020	Chen et al.	ROR1-AS1	↑	65			√	√	√	31929567
2020	Zhan et al.	SOX2OT	↑	106			√	√		32019566
2020	Luo et al.	TMPO-AS1	↑	40		√	√		√	32087328
2020	Xiong et al.	NRON	↑	42				√		32194786
2020	Wang et al.	BCAR4	↑	38				√	√	32273720
2020	Liu et al.	LINC00675	↓	89				√		32367602
2020	Wu et al.	ZNFX1-AS1	↑	67		√			√	32432735
2020	Dai et al.	SNHG3	↑	70		√		√		32596993
2020	Han et al.	TINCR	↑	71			√	√		32622721
2020	Li et al.	PVT1	↑	98			√	√		32664121
2020	He et al.	RBAT1	↑	30					√	32669100
2020	Zhan et al.	CASC9	↑	106			√	√		32677984
2020	Li et al.	IGFBP4-1	↑	100			√	√		32760196
2020	Li et al.	KCNQ1OT1	↑	30			√	√		32820233
2020	Xiang et al.	SNHG1	↑	60		√		√	√	32885590
2020	Wang et al.	SNHG7	↑	60					√	32898531
2020	He et al.	TMPO-AS1	↑	40				√		32964962
2020	Gui et al.	AFAP1-AS1	↑	40				√		32964963
2020	Yuan et al.	CASC9	↑	35				√		32982303
2020	Xu et al.	TINCR	↑	53				√		33000269
2020	Shen et al.	MAGI2-AS3	↓	80		√	√		√	33104021
2020	Zhang et al.	PCAT6	↑	106		√		√		33142195
2020	Chen et al.	PVT1	↑	70			√	√		33188158
2020	Huang et al.	CARLo-7	↑	143			√	√	√	33209690
2020	Tang et al.	MAGI2-AS3	↓	45				√		33231563
2020	Li et al.	MAFG-AS1	↑	43		√	√	√		33238264
2020	Kang et al.	PlncRNA-1	↑	28	√	√		√		33288752
2020	Liu et al.	FAM83H-AS1	↑	82				√		33289601
2020	Xiao et al.	MAFG-AS1	↑	102					√	33377647
2021	Zhang et al.	CASC9	↑	49	√			√		33200222
2021	Feng et al.	SNHG14	↑	62				√	√	33482820

The risk of tumor development in BCa varies according to the patient’s age and sex [187]. Interestingly, lncRNAs have no relationship with patient sex, while two studies have reported that CASC9 and PlncRNA-1 are associated with patient age. CASC9 upregulation is significantly positively correlated with BCa tumor invasion depth, histological grade, and age; however, sex and tumor volume were not related to CASC9 expression levels [62, 102, 148].

For BCa tumor size, several lncRNAs are related. The high expression level of ZNFX1-AS1 is related to advanced clinical stages and tumor size [47]. High expression of ZEB2-AS1 and SNHG5 is significantly correlated with tumor size, lymph node metastasis, and clinical stage [60, 93]. Patients with advanced-stage disease have higher levels of OIP5-AS1 expression than those with early-stage disease. High OIP5-AS1 expression is also observed in muscular invasion or large tumors [94]. Similarly, increased SNHG1 expression is closely correlated with tumor size, stage, invasion, and metastasis [188]. CCAT1 is positively related to clinical stage, tumor grade, and tumor size [69]. Increased ARSR expression is positively correlated with higher histological grade and larger tumor size [146]. SNHG3 [137], RMRP [132], PCAT6 [189], and LSINCT5 [149] expression positively correlated with tumor size and TNM stage, while high expression of MAFG-AS1 [118, 153], SNHG7 [90, 103], and TMPO-AS1 [33, 34] was closely related to histological grade, tumor size, TNM stage, and clinical stage of BCa patients. However, many lncRNAs are important for tumor size suppression. CASC2a is highly negatively correlated with pathological T and N stages, and tumor size [190]. HCG22 expression correlates with pathological stage, metastasis, and a large tumor range [26]. The MBNL1-AS1 expression level correlates with the clinical stage, tumor size, and focal classification [55]. In addition, downregulated MAGI2-AS3 correlates with the number of tumors, stage, grade, and stage [125, 150].

Accumulating evidence has revealed that the TNM stage, grade, and clinical/pathological stage of BCa can reflect the status of tumor development. High expression of HOTAIR and CDKN2B-AS is associated with a worse tumor grade. In addition, high expression of 5 lncRNAs positively correlates with tumor stage [21, 23, 37, 53, 54], while higher expression of 11 other lncRNAs is related to worse TNM stage [52, 61, 68, 70, 98, 110, 128, 156, 162, 182]. The expression of 4 lncRNAs is positively associated with an advanced disease stage and poor tumor grade [85, 154, 191]. Higher expression levels of 6 lncRNAs are associated with high tumor grade and advanced TNM stage [42, 108, 134, 185, 192, 193], while some other 4 lncRNAs are significantly correlated with T stage or metastasis, in addition to tumor grade [27, 36, 119, 175]. The expression level of 4 lncRNAs positively correlates with tumor progression stage and TNM stage [59, 88, 184, 194]. The expression of SNHG16 [83], BCAR4 [32], and SLCO4A1-AS1 [45] is related to metastasis and pathological stage. In addition, the high expression levels of LINC01296 [155], Carlo-7 [75], and ROR1-AS1 [54] are correlated with advanced tumor stage, higher tumor grade, and metastasis. In contrast, the expression of MIR143HG and MIR503HG is negatively correlated with tumor grade, advanced stage, and lymph node metastasis [111, 151]. Decreased expression of GAS5 [195] and DBCCR1-003 [196] is observed in BCa patients with higher grades, while LINC00675 [112] expression is decreased in lymph node-metastatic MIBC tissues compared to those without lymph node metastasis. Decreased expression of other lncRNAs, such as LBCS [186], MEG3 [77], and TP73-AS1 [152], is strongly associated with tumor stage, grade, and/or TNM stage.

## LncRNAs that influence patient prognosis

Some lncRNAs can be used to predict patient prognoses, such as overall survival (OS), disease-free survival (DFS), recurrence-free survival (RFS), and progression-free survival (PFS). Here, we reviewed the survival data from studies relating to BCa to determine the prognostic value of lncRNAs, in terms of OS, DFS, RFS, and PFS. In the last 10 years, more than 60 lncRNAs with the potential to predict patient prognosis have been reported (Table 3). Among them, 3 lncRNAs downregulated in BCa have been found to predict poor PFS [24, 152, 195], whereas 4 lncRNAs upregulated in BCa predict poor PFS [23, 40, 50, 189]. The results of prognosis analysis revealed that high expression of CASC9 [102, 148], SNHG3 [137], and SOX2OT [185], and low expression of LBCS [186] predict poor DFS. Elsewhere, high expression of CASC2a [190] increased the 5-year RFS rate, and high expression of 8 lncRNAs predicted a low RFS rate [21, 23, 37, 38, 115, 146, 156, 164, 188, 193]. In addition, lower expression of 7 lncRNAs predicted shorter OS [24, 26, 57, 126, 150, 152, 186]. High expression of 28 lncRNAs predicted shorter OS [23, 27, 32, 39, 40, 45, 50, 53, 60, 83, 94, 100, 118, 121, 123, 128, 130, 137, 141, 146, 155, 156, 162, 164, 185, 192, 194, 197].

**Table 3:** The relationship between LncRNA and prognosis.

LncRNA	Expression	Prognostic	OS	PFS	DFS	RFS	Ref./PMID
CASC2a	↓	√				√	29358570
DGCR5	↓	√					30238982
GAS5	↓	√		√			27878359 30374124
HCG18	↓	√	√				30426533
HCG22	↓	√	√				31304601
LBCS	↓	√	√		√		30397178
LINC00641	↓	√	√	√			30060954
LINC00675	↓	√					32367602
MAGI2-AS3	↓	√	√				30442369 33104021
MIR143HG	↓	√					30471109
PLAC2	↓	√	√				32650766
TP73-AS1	↓	√	√	√			29625110
AFAP1-AS1	↑	√					32964963
ARAP1-AS1	↑	√	√				30404578
ASAP1-IT1	↑	√					28895409
BCAR4	↑	√	√				32273720
CALML3-AS1	↑	√	√	√			30177388
CASC9	↑	√			√		32677984 33200222
DLEU1	↑	√					30984249
DLX6-AS1	↑	√	√				31615303 32756011
EGFR-AS1	↑	√	√				32194685
ELF3-AS1	↑	√	√				30528231
FAM83H-AS1	↑	√	√				30537032 33289601
FOXD2-AS1	↑	√	√	√		√	29445134
GClnc1	**↑**	√					31298933
HNF1A-AS1	↑	√	√				29762827
HOTAIR	↑	√					26781446
HULC	↑	√				√	28946549
IGFBP4-1	↑	√	√				32760196
ITGB1	↑	√	√				31486485
LINC00162	↑	√					33344916
LINC00319	↑	√				√	31608995 32194636
LINC00460	↑	√					30881506
LINC00857	↑	√	√			√	29856124
LINC01140	↑	√	√				33234721
LINC01296	↑	√	√				30588032
LINC01605	↑	√					30054424
ARSR	↑	√	√			√	31892841
n336928	**↑**	√	√				26551459
LSINCT5	↑	√					29772237
MAFG-AS1	↑	√	√				33238264 33377647 33400245
MALAT1	↑	√	√				24449823 29736319
n346372	**↑**	√					30365104
NCK1-AS1	↑	√					32184669
NRON	↑	√	√			√	32194786
OIP5-AS1	↑	√	√				30485498
PCAT6	↑	√	√	√			33090394 33142195
PVT1	↑	√					32664121
RMRP	↑	√					30779067
RNF144A-AS1	↑	√					33177836
ROR1-AS1	↑	√					31929567
SLCO4A1-AS1	↑	√	√				30863101
SNHG1	↑	√				√	32885590
SNHG14	↑	√					33482820
SNHG16	↑	√	√				29234154
SNHG20	↑	√					30106094
SNHG3	↑	√	√		√		32596993
SNHG5	↑	√	√				29434891
SNHG7	↑	√					30527358 32898531
SOX2OT	↑	√	√		√		32019566
TINCR	↑	√				√	32622721 33000269
TMPO-AS1	↑	√					32087328 32964962
TUG1	↑	√	√				26318860 30925453
XIST	↑	√					31602223
ZFAS1	↑	√	√	√			29653362

## Conclusion

**Fig. 2:**
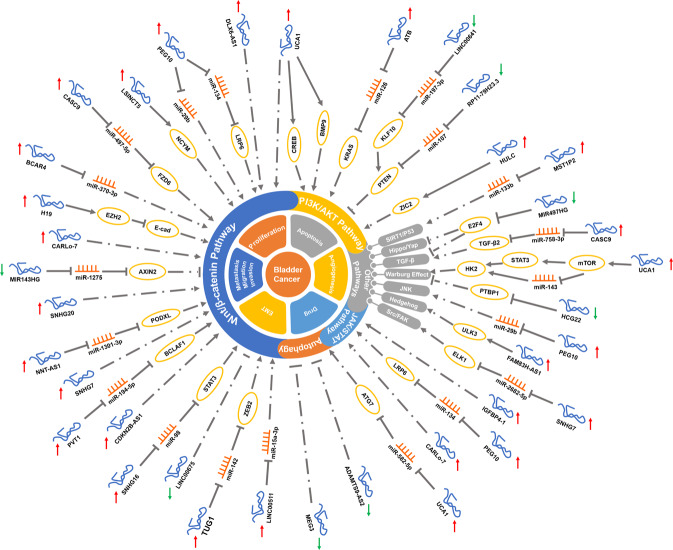
The detailed mechanisms of lncRNAs on tumor classic pathways in BCa.

Researchers have already found that more than hundreds of lncRNAs could affect the initiation and progression of BCa. In the past 10 years, several biological functions of lncRNAs have been reported, especially in the past two years. As described in this review, more than 100 lncRNAs influence the proliferation, apoptosis, invasion, migration, metastasis, drug resistance, and even CSCs in BCa. Other BCa-related lncRNAs can act as ceRNA regulatory mechanisms to regulate various processes in tumors (Fig. 2). The studies reviewed here also indicate that lncRNAs are strongly associated with BCa patients’ clinicopathological characteristics and prognosis, demonstrating that lncRNAs may be potential diagnostic and prognostic biomarkers for BCa patients.

Several questions remain regarding the role of lncRNAs in BCa. Evidence indicates that one lncRNA can regulate more than one gene. The relationship between such genes should be further investigated. Apart from acting as miRNA sponges and via ceRNA mechanisms, other important mechanisms, such as ubiquitination and other posttranscriptional modifications, should be studied. Moreover, clinical studies with a large sample should be designed to explore the roles of lncRNAs in BCa from the perspectives of epigenetics and posttranscription. In addition, multicenter cohort studies are necessary to validate the diagnostic, prognostic and therapeutic value of lncRNAs in BCa.

## Data Availability

All data generated or analyzed during this study are included in this published article.
